# Case Report: Recombinant Human Endostatin Plus Chemotherapy for Epidermal Growth Factor Receptor-Negative Miliary Lung Adenocarcinoma

**DOI:** 10.3389/fonc.2022.922076

**Published:** 2022-07-04

**Authors:** Jian Zhu, Ya Xu, Wen-Cai Huang, Tao Ji, Guo-Ping Ai, Yan-Hong Gao

**Affiliations:** ^1^ Department of Thoracic Cardiovascular Surgery, General Hospital of Central Theater Command of the People’s Liberation Army, Wuhan, China; ^2^ Department of Respiratory Medicine, General Hospital of Central Theater Command of the People’s Liberation Army, Wuhan, China; ^3^ Department of Radiology, General Hospital of Central Theater Command of the People’s Liberation Army, Wuhan, China; ^4^ Department of Ultrasound, General Hospital of Central Theater Command of the People’s Liberation Army, Wuhan, China

**Keywords:** lung adenocarcinoma, miliary metastasis, immunotherapy, anti-angiogenic therapy, recombinant human endostatin

## Abstract

Except for the traditional chemotherapy, few treatments strategy about miliary intrapulmonary carcinomatosis (MIPC) have been reported in the existing literature. In this report, we primarily discussed the possible etiology and the potentially effective treatment options for a patient with MIPC who benefited from combined treatment. A nonsmoking woman was diagnosed with MIPC at an advanced stage. Gene detection showed an EGFR negative status. She accepted first-line chemotherapy with pemetrexed and cisplatin, and the tumor progressed. Next, PD-1 inhibitors plus pemetrexed and cisplatin were administered, and the tumor remained uncontrolled. After two cycles of recombinant human endostatin plus second-line chemotherapy, the numerous pulmonary nodules had all nearly completely disappeared, while an accentuated decrease in the primary tumor volume was observed. Moreover, biochemical markers, including the patient’s tumor markers, also trended toward normal. This report describes the first case of a MIPC patient who significantly responded to antiangiogenic therapy combined with chemotherapy. Anti-angiogenic therapy may be a possible strategy for the EGFR-negative lung adenocarcinoma population.

## Introduction

Miliary intrapulmonary carcinomatosis (MIPC) is a rare lung cancer presentation characterized by diffuse, tiny and discrete pulmonary micronodules (1). Typical metastatic miliary nodules of lung cancer is rarely seen in the literature. MIPC may occur at initial diagnosis or during the progression of the disease after treatment (2). Moreover, there is currently no clearly established consensus on the treatment of the disease. We present a case of MIPC with classical miliary lung nodules on CT images. After treatment failure of first-line chemotherapy and immunotherapy, she achieved dramatic efficacy from two cycles of chemotherapy combined with anti-angiogenic therapy. The excellent response included an almost complete disappearance of miliary lung metastasis, a decrease in her tumor markers, and relief of her symptoms. Through study of the treatment process of this case, some enlightenment may be achieved for understanding the possible etiology of this disease and it provides a potentially effective treatment option for patients with MIPC.

## Case Report

**Figure 1:**
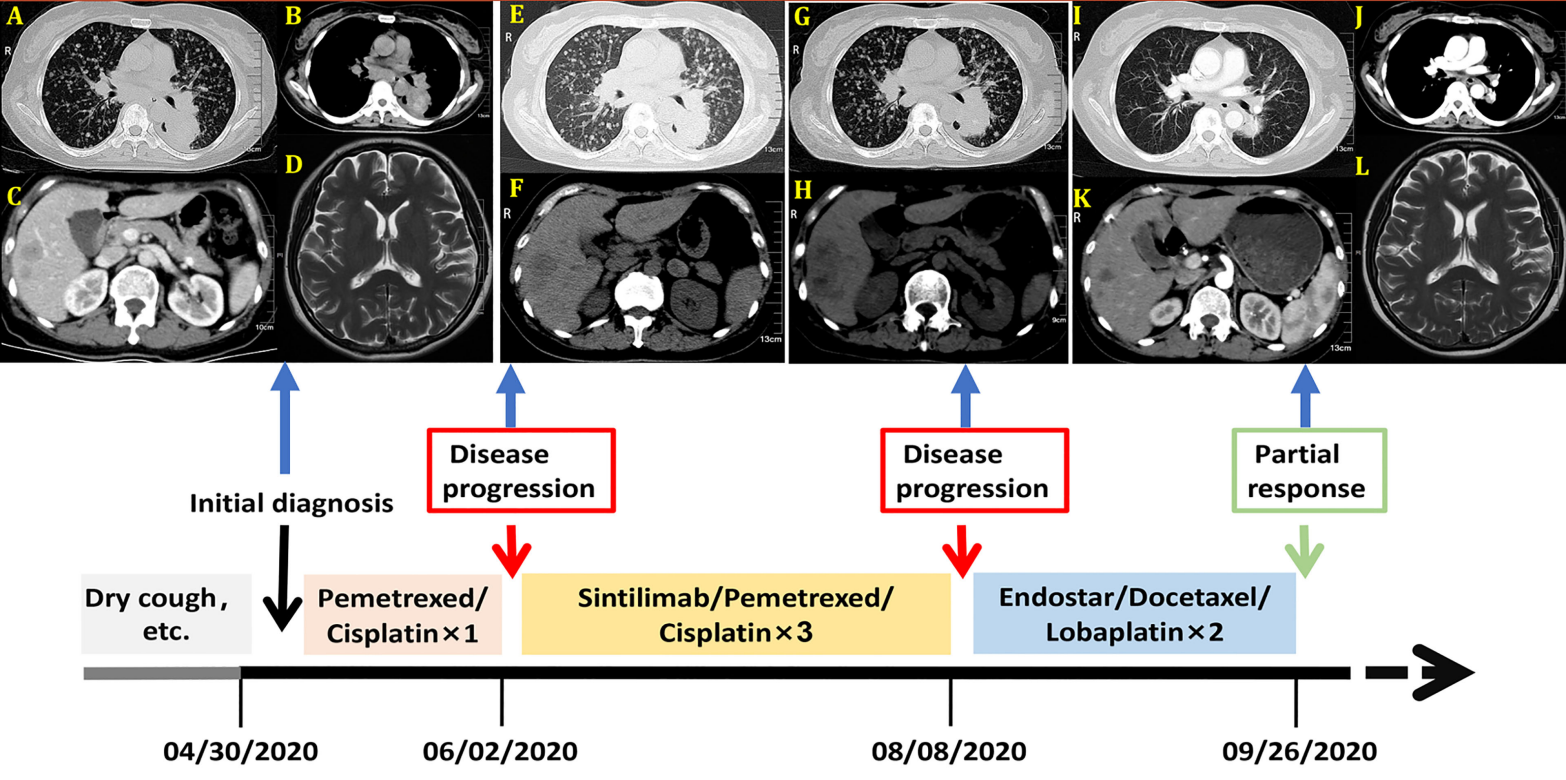
Imagines of computed tomography (CT) and Magnetic resonance (MR) before treatment. **(A)** Chest CT scan with lung window; **(B)** Chest CT scan with mediastinal window; **(C)** Abdominal-enhanced CT scan; **(D)** Head magnetic resonance (MR) image. Imagines of CT after the first cycle of pemetrexed and cisplatin. **(E)** Chest CT scan with lung window; **(F)** Abdominal CT scan; Imagines of CT after three cycles of Sintilimab combined with pemetrexed and cisplatin. **(G)** Chest CT scan with lung window; **(H)** Abdominal CT scan; Imagines of computed tomography (CT) and Magnetic resonance (MR) after two cycles of Endostar combined with docetaxel and lobaplatin. **(I)** Chest CT scan with lung window; **(J)** Chest CT scan with mediastinal window; **(K)** Abdominal-enhanced CT scan; **(L)** Head magnetic resonance (MR) image. These imagines were showed the timeline of different treatments for the patient’s entire treatment progression.

**Figure 2:**
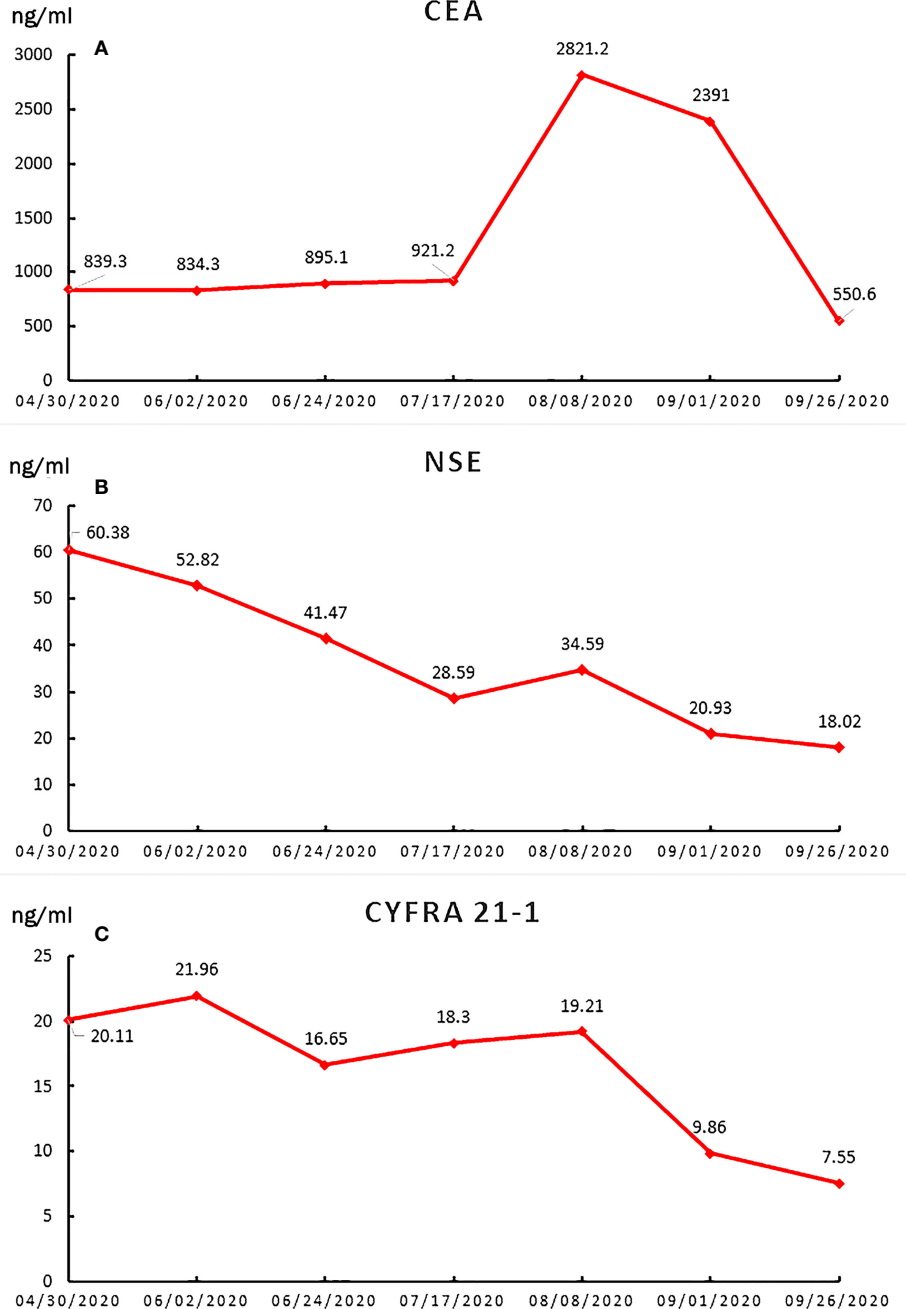
The line charts of tumor biomarkers during the treatment period. **(A)** CEA; **(B)** NSE; **(C)** CYFRA21-1.

A 57-year-old nonsmoking woman complained of cough, shortness of breath, chest distress, dyspnea, thoracolumbar and back pain for nearly one month and presented to our center just after the end of the COVID-19 lockdowns in Wuhan. She denied a history of tuberculosis exposure and any other history of disease except for well-controlled hypertension. She was notable for apparent bilateral supraclavicular lymphadenectasis after a physical examination. A chest computed tomography (CT) scan revealed numerous uniform pulmonary nodules in the bilateral lungs in addition to a 38x45 mm mass at the dorsal segment of the lower lobe of the left lung, with bilateral hilar and mediastinal lymphadenopathy (**Figures 1A, B**), and multiple bone metastases (including thoracic spine, sternum, ribs and left scapula). The enhanced CT scans showed multiple liver (the largest nodule about 21x21mm) and adrenal metastases (**Figure 1C**). Magnetic resonance examination of the head revealed no obvious metastases (**Figure 1D**). Her laboratory results (complete blood cell count: WBC (white blood cell) 8.6×10^9^/L, RBC (red blood cell) 4.08×10^9^/L, PLT (platelet) 433×10^9^/L, liver panel: ALT (alanine aminotransferase) 19U/L, AST (aspartate aminotransferase) 34U/L, kidney panel: creatinine 47μmol/L) were within normal limits, her respiratory etiology test results (including tuberculosis) were negative, but her tumor markers were elevated (**Figure 2**).

**Figure 3:**
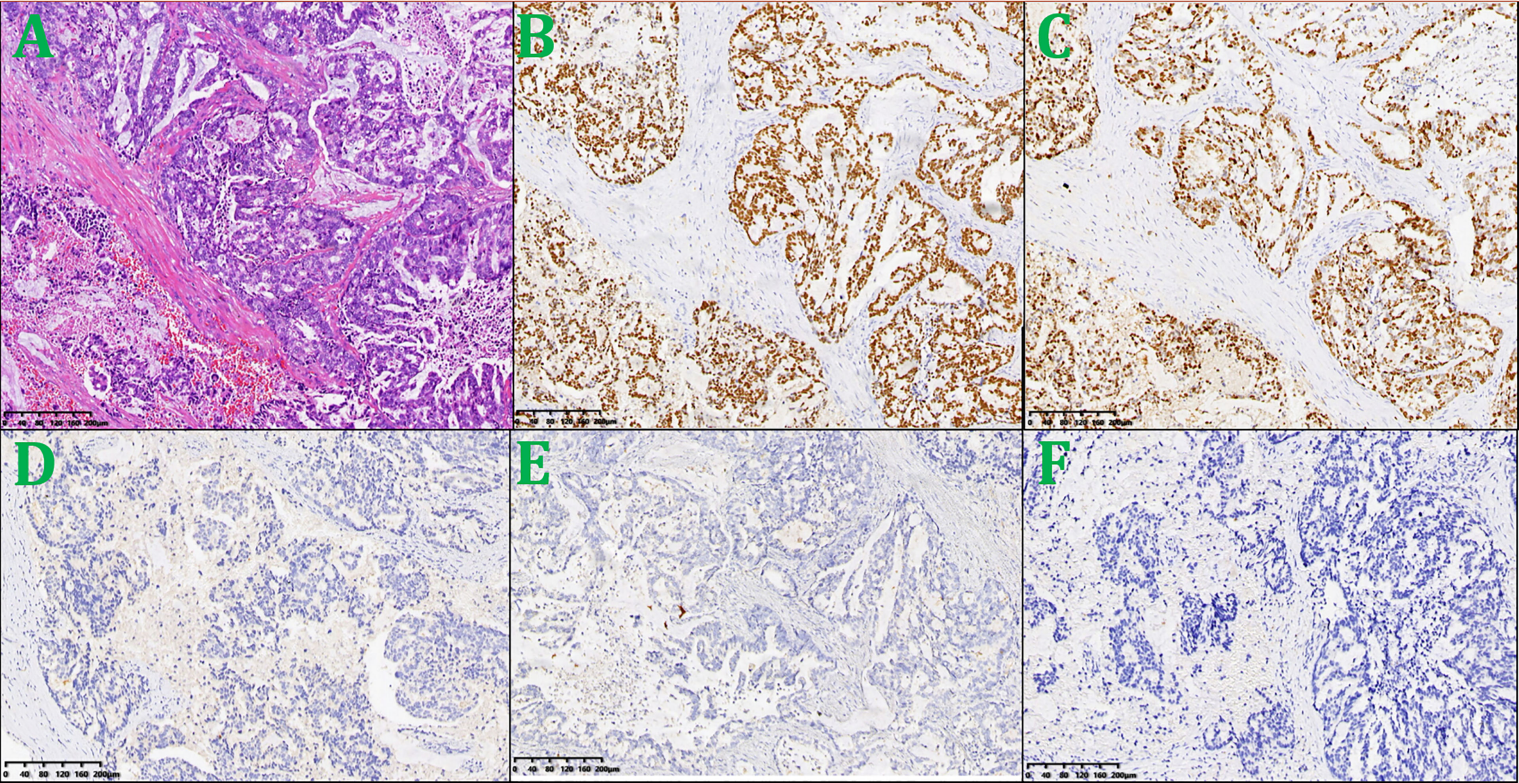
Lesion biopsy specimen of the left supraclavicular lymph node. **(A)** HE × 100; **(B)** TTF-1× 100; **(C)** Ki-67 × 100; **(D)** Syn × 100; **(E)** P40 × 100; **(F)** PD-L1 × 100.

A supraclavicular lymph node biopsy was performed. Immunohistochemical staining was positive for PCK, CK7, TTF-1, and Ki-67 at 70%; negative for P40, Napsin A, Syn, and CDX-2; PD-L1(=1%, Clone: 22C3, Dako, Agilent Technologies, Inc.); which is consistent with lung adenocarcinoma (**Figure 3**). Genetic testing of paraffin section from supraclavicular lymph node was conducted in CLIA-certified lab using hybridization capture-based next-generation sequencing panels. Gene targets (MyGene, BGI-Shenzhen [Headquarters]: Shenzhen, 518083, China. Genomic alterations assessed included single nucleotide variations, insertions and deletions, copy number variations, and gene rearrangements in selected genes. The MyGene panels covered common lung cancer-related genes. The minimum coverage across sample was ≥1000×. Actionability of genomic alterations and the level of evidence were determined based on the OncoKB data set and drug approval status in mainland China (3)] revealed no mutated genes as EGFR [Exon 18/19/20/21/T790), FGFR2, FGFR3, ROS1, TP53, RET, PIK3CA (Exon 10/12, coding exon 9/20), ALK, KRAS (Codon 12/13/61/146), NRAS (Codon 12/13/61), KIT (Exon 9/11), BRCA1, BRCA2, and ERBB2 (Exon 20/copy number amplification). She was diagnosed with lung adenocarcinoma cT4N3M1 stage IVb, EGFR-negative (According to the eighth edition TNM classification, innumerable nodules in the ipsilateral lobes are categorized as T4, and those in a contralateral lobe while multiple metastases in distant organ are M1, bilateral hilar and mediastinal lymphadenopathy are N3).

She was given a cycle of pemetrexed (day 1: 500mg/m^2^) and cisplatin (day 1-3: 25mg/m^2^ every day), but reexamination of her chest CT and abdominal CT showed no signs of a reduction in her pulmonary nodules (**Figures 1E, F**). Then, three cycles of sintilimab (PD-1 inhibitor, day 1: 200mg) plus pemetrexed (day 1: 500mg/m^2^) and cisplatin (day 1-3: 25mg/m^2^ every day) were administered, the patient suffered greatly thoracolumbar and back pain, fierce cough, wheezing, chest distress, and dyspnea. Although there were no other immune-related adverse events, the treatment was terminated because of persistent disease progression (**Figures 1G, H**). Then, the patient received two cycles of recombinant human endostatin (Endostar, day 1-14: 7.5mg/m^2^ every day) combined with docetaxel (day 1: 75mg/m^2^) and lobaplatin (day 1: 75mg/m^2^). Her white blood cells were normal, liver and kidney function tests were normal (WBC 5.4×10^9^/L, RBC 3.53×10^9^/L, PLT 313×10^9^/L, ALT 25U/L, AST 33U/L, creatinine 39μmol/L). Her symptoms, including cough, shortness of breath, wheezing, chest distress, dyspnea, thoracolumbar and back pain, were significantly relieved. She had no drug-related symptoms other than mild itching on his skin during this treatment period. Fortunately, chest and abdomen CT scans revealed almost complete disappearance of all pulmonary nodules, as well as an accentuated decrease in the primary lung mass (32x32mm) and liver metastasis volume (the largest one about 16x15mm) after this two cycle’s treatment (**Figures 1I–L**). The diagram of her specific treatment progress see **Figure 1**.

## Discussion

Tuberculosis is the most common cause of miliary nodules (4). Histoplasmosis, sarcoidosis, pneumoconiosis, bronchoalveolar carcinoma, pulmonary siderosis, and hematogenous metastases from primary cancers (mainly thyroid, kidney, trophoblast, and some of the sarcomas) are the relatively rare etiologies that have been described. MIPC is a rare lung cancer presentation characterized by diffuse, tiny and discrete pulmonary micronodules (1). MIPC may occur at initial diagnosis or during the progression of the disease after treatment (2). Patients with MIPC have higher rates of EGFR mutations (the exon 19 deletion in particular) and adenocarcinoma, especially in never-smoking Asian patients (5, 6). EGFR tyrosine kinase inhibitors are recommended as the treatment of choice for non-small cell lung cancer (NSCLC) patients with MIPC at initial diagnosis among Asians (5). These suggestions may be further supported by the notion that MIPC is much less common in patients who have lung adenocarcinomas with wild-type EGFR (7). However, our case is a never-smoking Chinese female adenocarcinoma patient with MIPC at initial assessment, consistent with previous study findings, except without EGFR mutations. NSCLC patients presenting with MIPC have a rapid progression course (5) and poor outcomes (8). Unfortunately, the only previous report showed that an EGFR-negative MIPC patient suffered a much worse outcome (9).

In our case, the patient lost the chance of surgical treatment because the primary tumors were widely disseminated to multiple distant organs and lymph nodes at the time of initial diagnosis. We detected a series of possible lung cancer mutation sites after an extensive literature review, but the results of her genetic tests rejected any consideration of targeted therapy. Chemotherapy is an accessible classical standard regimen. Due to the lack of a specific treatment strategy for EGFR-negative MIPC, we used PD-1 inhibitor as a tentative solution. This is not without reason, of course. Immunotherapy (including sintilimab) and chemotherapy can significantly extend the progression‐free survival period of NSCLC patients (10). Thus, chemotherapy plus immunotherapy was considered, but the subsequent examination showed disease progression. Then, we recommended antiangiogenic therapy combined with chemotherapy. Endostar, a broad-spectrum angiogenesis inhibitor, was approved by the China Food and Drug Administration (CFDA) for the treatment of NSCLC based on significantly improved treatment outcomes (11). The patient responded remarkably to this combination treatment. The miliary lung metastasis disappeared almost completely, accompanied by a significant reduction in serum tumor markers and significant relief of the patient’s symptoms, which mainly including cough, shortness of breath, chest distress, dyspnea, thoracolumbar and back pain.

This patient experienced a bad situation after the failure of first-line chemotherapy and PD-1 inhibitors combined with chemotherapy. The patient suffered greatly thoracolumbar and back pain, fierce cough, wheezing, chest tightness, and dyspnea. Imaging and serum markers rapid progression might objective indicated a extremely worse situation of the patient. Fortunately, two cycles of combined antiangiogenic therapy with chemotherapy have achieved spectacular success in the absence of a better choice. After 40 days, the patient’s disease was remarkably relieved, reflecting remarkable amelioration of her symptoms, sharply decreasing tumor markers and a significant radiographic response. Undoubtedly, the antiangiogenic therapy is the major strength of the anticancer effect along with some effect from the chemotherapy.

The reasons for this surprising treatment response in our patient may be of the utmost relevance because of the abundant neovascularization and the recognized metastatic pathway. Endostar inhibits vascular endothelial cell proliferation, normalizes the tumor vasculature, and alleviates tumor microenvironment hypoxia, thereby suppressing angiogenesis and tumor growth (12). MIPC is generally considered to be a result of tumor cell hematogenous dissemination. MIPC in lung cancer has been associated with bone metastasis (13). Patients presenting with MIPC tend to have metastases in multiple bones and distant organs, all consistent with the characteristics of hematogenous routes (5). These statuses are consistent with the baseline situation of our report. Additionally, lung adenocarcinoma is more closely associated with angiogenesis than squamous cell carcinoma (14), which may further contribute to the high potential of hematogenous spread (15). These findings support a close link between hematogenous spread and MIPC. However, the specific associations and mechanisms between the above and EGFR mutations in NSCLC need to be investigated further.

## Conclusions

In conclusion, we must admit that NSCLC patients who benefit from immunotherapy are limited in the current immunotherapy era. This case with individualizing treatment indicated that combining antiangiogenic therapy with chemotherapy might be an effective therapeutic regimen for miliary lung adenocarcinoma patients, especially in the cancer with the abundant neovascularization and hematogenous metastasis. Additionally, this report shows a more elegant picture about numerous uniform pulmonary nodules (like many pearls) in the bilateral lungs.

## Data Availability Statement

The original contributions presented in the study are included in the article/supplementary material. Further inquiries can be directed to the corresponding authors.

## Ethics Statement

Written informed consent was obtained from the participant(s) for the publication of this case report.

## Author Contributions

W-CH and YX offered the case and collected the data. JZ prepared the manuscript. G-PA and Y-HG revised the manuscript. All authors contributed to have agreed on the journal to which the article will be submitted, gave final approval of the version to be published, and agree to be accountable for all aspects of the work.

## Funding

This paper is supported by grants from Yuying Plan with Growth Project of General hospital of central theater command of the People’s Liberation Army, CHINA [No. ZZYCZ202106] and Primary Research & Development Plan of Hubei Province, CHINA [No. 2020BCB059].

## Conflict of Interest

The authors declare that the research was conducted in the absence of any commercial or financial relationships that could be construed as a potential conflict of interest.

## Publisher’s Note

All claims expressed in this article are solely those of the authors and do not necessarily represent those of their affiliated organizations, or those of the publisher, the editors and the reviewers. Any product that may be evaluated in this article, or claim that may be made by its manufacturer, is not guaranteed or endorsed by the publisher.

## Abbreviations

CT, computed tomography; NSCLC, non-small-cell lung cancer; PD-1, programmed cell death-1; MIPC, miliary intrapulmonary carcinomatosis; COVID-19, coronavirus disease 2019; EGFR, epidermal growth factor receptor; CFDA, China Food and Drug Administration.
